# Evaluation of early antimicrobial therapy adaptation guided by the BetaLACTA® test: a case-control study

**DOI:** 10.1186/s13054-017-1746-6

**Published:** 2017-06-28

**Authors:** Marc Garnier, Sacha Rozencwajg, Tài Pham, Sophie Vimont, Clarisse Blayau, Mehdi Hafiani, Jean-Pierre Fulgencio, Francis Bonnet, Jean-Luc Mainardi, Guillaume Arlet, Muriel Fartoukh, Salah Gallah, Christophe Quesnel

**Affiliations:** 1 Département d’Anesthésie et Réanimation, APHP Hôpital Tenon, 4 rue de la Chine, 75020 Paris, France; 2 APHP Hôpital Tenon, Unité de Réanimation Médico-Chirurgicale, Paris, France; 3 Université Pierre et Marie Curie Sorbonne Université, Paris, France; 4 Interdepartmental Division of Critical Care Medicine, University of Toronto, Toronto, Canada; 5 Keenan Research Centre, Li Ka Shing Knowledge Institute, St Michael’s Hospital, Toronto, Canada; 6 Département de Bactériologie, APHP Hôpitaux Universitaires Est Parisiens - site Tenon, Paris, France; 7 APHP Hôpital Européen Georges Pompidou, Service de Microbiologie, Paris, France; 8 Université Paris Descartes, Paris, France

**Keywords:** Antimicrobial agent administration, Microbial susceptibility tests, Beta-lactam resistance, Extended-spectrum beta-lactamase, Intensive care unit

## Abstract

**Background:**

Rapid diagnostic tests detecting microbial resistance are needed for limiting the duration of inappropriateness of empirical antimicrobial therapy (EAT) in intensive care unit patients, besides reducing the use of broad-spectrum antibiotics. We hypothesized that the betaLACTA® test (BLT) could lead to early increase in the adequacy of antimicrobial therapy.

**Methods:**

This was a case-control study. Sixty-one patients with BLT-guided adaptation of EAT were prospectively included, and then matched with 61 “controls” having similar infection characteristics (community or hospital-acquired, and source of infection), in whom EAT was conventionally adapted to antibiogram results. Endpoints were to compare the proportion of appropriate (primary endpoint) and optimal (secondary endpoint) antimicrobial therapies with each of the two strategies, once microbiological sample culture results were available.

**Results:**

Characteristics of patients, infections and EAT at inclusion were similar between groups. Nine early escalations of EAT occurred in the BLT-guided adaptation group, reaching 98% appropriateness vs. 77% in the conventional adaptation group (*p* < 0.01). The BLT reduced the time until escalation of an inappropriate EAT from 50.5 (48–73) to 27 (24–28) hours (*p* < 0.01). Seventeen early de-escalations occurred in the BLT-guided adaptation group, compared to one in the conventional adaptation group, reducing patients’ exposure to broad-spectrum beta-lactam such as carbapenems. In multivariate analysis, use of the BLT was strongly associated with early appropriate (OR = 18 (3.4–333.8), *p* = 0.006) and optimal (OR = 35.5 (9.6–231.9), *p* < 0.001) antimicrobial therapies. Safety parameters were similar between groups.

**Conclusions:**

Our study suggests that a BLT-guided adaptation strategy may allow early beta-lactam adaptation from the first 24 hours following the beginning of sepsis management.

**Electronic supplementary material:**

The online version of this article (doi:10.1186/s13054-017-1746-6) contains supplementary material, which is available to authorized users.

## Background

Sepsis remains a major cause of mortality among patients admitted to the Intensive Care Unit (ICU) [1]. Among determinants of sepsis-related death, the adequacy of empirical antimicrobial therapy (EAT) and the rapidity of its initiation are the most important protective factors [2, 3]. Due to the increased incidence of extended-spectrum beta-lactamases (ESBL)-producing Gram-negative bacilli (GNB), carbapenem consumption has rapidly increased worldwide, reaching for instance +30% in French ICUs between 2008 and 2011 [4]. This in turn favours the emergence of carbapenemase-producing *Enterobacteriaceae* [5]. To minimize the individual risk of an inappropriate EAT and at the same time the societal risk of excessive broad-spectrum beta-lactams consumption, early initiation of broad-spectrum EAT is recommended, followed by its de-escalation as soon as the results of antibiotic susceptibility tests (AST) are available [6, 7]. However, this conventional AST-guided de-escalation occurs 48 to 72 hours after initiation of EAT [8]. On one hand, this time is too long for the 10–30% of patients treated with inappropriate EAT [3, 9–11]; on the other hand, this time is sufficient for the carriage of carbapenem-resistant bacteria to emerge [12]. Thus, strategies for limiting the duration of EAT inadequacy, at the same time as reducing the use of broad-spectrum beta-lactam antibiotics are urgently needed [13].

The betaLACTA® test (BLT) is a chromogenic diagnostic device detecting resistance of GNB to 3^rd^ generation cephalosporins (3GC) due to β-lactamases from ESBL, carbapenemases or acquired AmpC classes, in less than 20 minutes. The BLT showed interesting intrinsic diagnostic performance in laboratory evaluations, notably sensitivity and specificity >95% for ESBL detection on *Enterobacteriaceae* colonies [14–17]. However, to our knowledge, a BLT-guided antimicrobial adaptation strategy has never been investigated in the clinical setting.

We hypothesized that a BLT-guided strategy could lead to earlier improvement in the adequacy of EAT compared to a conventional strategy in ICU patients with *Enterobacteriaceae*-related infection (i.e. escalation in the case of inappropriateness or de-escalation in the case of treatment being too broad-spectrum). Our primary aim was to assess a BLT-guided strategy to improve the proportion of appropriate antimicrobial therapy once the results of the microbiological sample cultures were available. Our secondary aim was to assess a BLT-guided strategy to improve the proportion of optimal antimicrobial therapy.

## Methods

See “Additional methods” (Additional file 1).

### Ethical considerations

This case-control study was conducted following the Strengthening of Reporting in Observational studies in Epidemiology (STROBE) statements [18] and was approved by the Institutional Review Board (Comité de Protection des Personnes “Ile de France VI”, Paris, France, ID-RCB 2015-A00169-40), and by the French national commission on digital storage of personal data (CNIL, number 1832114). Information was given to patients or next of kin, who could decline inclusion in the study.

### Cases and controls selection

After BLT introduction as a routine susceptibility test in our laboratory in January 2014, all ICU patients were screened daily for eligibility as “cases”. Eligibility criteria were the presence of an active infection caused by at least one *Enterobacteriaceae* species and requirement of EAT. Exclusion criteria were: (1) antimicrobial therapy withdrawal within the first 48 hours of treatment and/or diagnosis reclassification from infection to colonisation, (2) existence of a concomitant infection preventing antimicrobial de-escalation or (3) a moribund patient or a patient in whom a procedure of withdrawing life-sustaining treatment was decided. “Cases” (i.e. patients with BLT-guided EAT adaptation) were consecutively and prospectively included among eligible patients. One “control” (i.e. a patient with AST-guided EAT adaptation), matched both on the site and the community-acquired or healthcare-associated status of the infection, was recruited per case among the last patients who presented an *Enterobacteriaceae* infection before introduction of the BLT.

### Empirical antimicrobial therapy

The composition of the EAT was prescribed according to local practice guidelines adapted from international guidelines (see Additional file 2). Cefotaxime was selected for community-acquired and early-onset nosocomial infections. Piperacillin-tazobactam, cefepime, imipenem-cilastatin, doripenem or meropenem were selected for late-onset nosocomial infections. An aminoglycoside was added in the case of septic shock or in high suspicion of *Pseudomonas aeruginosa*. ICU physicians and prescription guidelines did not change during the two study periods, except for withdrawal of doripenem from August 2014.

### Microbiological procedures

BLT was performed by a trained microbiologist on freshly isolated *Enterobacteriaceae* colonies as recommended by the manufacturer. Results were interpreted as positive if the colour turned red, purple or orange, and negative if the colour remained yellow. The cost of the device cost was 3.5 €. AST was performed by the disk diffusion method. If several *Enterobacteriaceae* were isolated in culture, the BLT and AST were performed on each species, and EAT was adapted considering the most resistant phenotype.

### Antimicrobial adaptation strategies

In the conventional adaptation group (i.e. the controls), empirical beta-lactam could be adapted to results of microbiological sample culturing, notably *Enterobacteriaceae* identification, and then definitively adapted to AST.

**Fig. 1:**
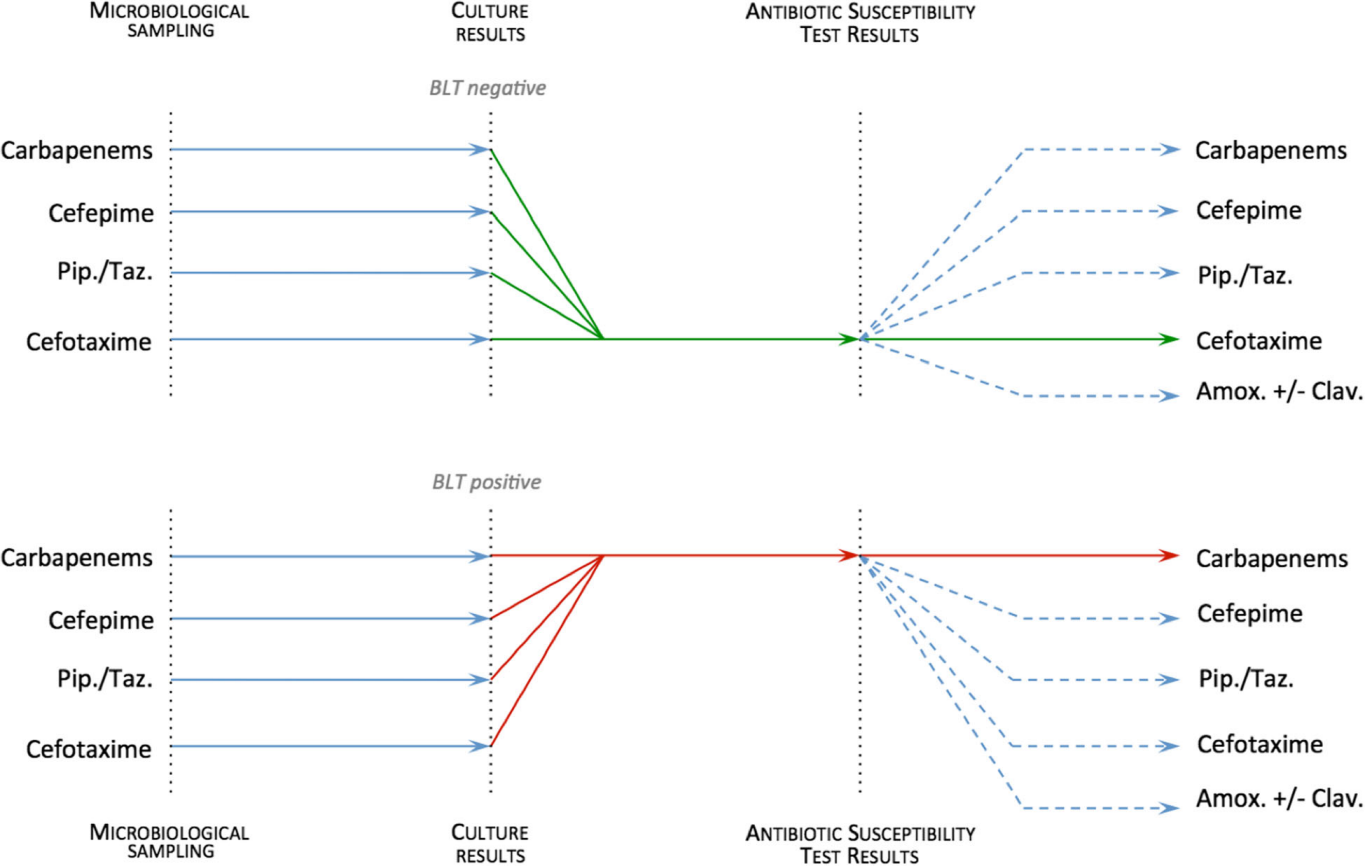
Empirical antimicrobial therapy adaptation in the case of a negative (*upper panel*) or positive (*lower panel*) betaLACTA® test (*BLT*). Whatever the beta-lactam empirically administered to patients, cefotaxime was chosen to pursue antimicrobial therapy once the BLT results were negative. Conversely, carbapenems were chosen if the BLT was positive. The definitive antimicrobial therapy was prescribed after the results of antibiotic susceptibility tests, confirming the BLT-adapted beta-lactam antibiotic or using another molecule if necessary. *Amox.* amoxicillin, *Clav.* clavulanate, *Pip.* piperacillin, *Taz.* tazobactam

In the BLT-guided adaptation group (i.e. the cases), empirical beta-lactam was adapted to the BLT results (Fig. 1). When needed, the attending physician could secondarily adapt the BLT-guided beta-lactam to the AST.

### Definitions and endpoints

An appropriate antimicrobial therapy was defined as use of a beta-lactam active on the identified *Enterobacteriaceae* as determined by the AST as the gold standard. According to the beta-lactam classes defined by Weiss and colleagues [19], an “optimal antimicrobial therapy” was defined as the use of amoxicillin, amoxicillin/clavulanate or cefotaxime for wild-type *Enterobacteriaceae;* cefepime for AmpC-overproducing *Enterobacteriaceae*; and carbapenem for ESBL-producing *Enterobacteriaceae*.

### Statistical analysis

The sample size estimation was based on the chi-square test for comparison of two proportions. Assuming a proportion of 80% of appropriateness of EAT in the conventional adaptation group [9–11, 20], 60 patients per group were needed to show an increase to a proportion of 95% of appropriateness of the antimicrobial therapy in the BLT-guided adaptation group, with alpha risk of 5% and power of 80%.

Differences in categorical variables were analysed using the chi-square or the Fisher exact test or the McNemar test for matched patients, as appropriate. Continuous variables were reported as median [interquartile range], and were compared using the matched-pairs Wilcoxon rank-sum test.

Further, multivariate analyses were performed to determine variables independently associated with appropriate and optimal antimicrobial therapies. Both covariates known to impact the choice of EAT determined a priori and those with *p* < 0.20 in bivariate analyses were entered into multivariate regression models with variable selection based on a stepwise backward and forward elimination procedure using *p* values. All included patients were considered for analysis. A *p* value <0.05 was considered as significant. Statistical analysis was performed using GraphPad Prism 6 (GraphPad Software, San Diego, CA, USA) and R3.3.2 (http://www.R-project.org).

## Results

### Patient characteristics

**Fig. 2:**
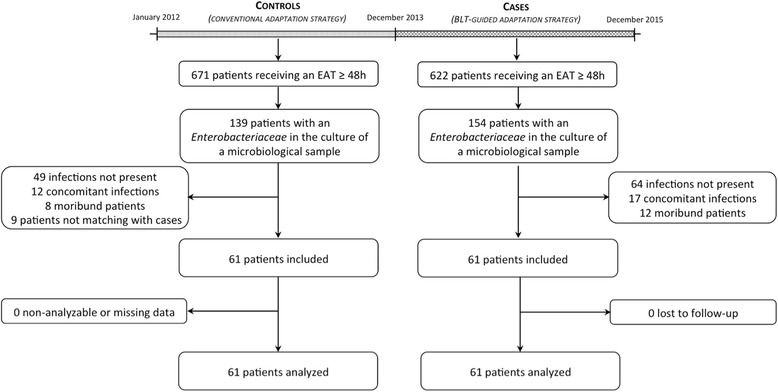
Flow chart of the study. During the betaLACTA test (*BLT*)-guided adaptation period, 622 patients receiving empirical antimicrobial therapy (*EAT*) were screened, among whom 61 cases were finally included. The comparative conventional adaptation group included 61 controls matched both on the site and the community-acquired or healthcare-associated status of the infection among the 671 patients having received EAT during the last 24 months before the introduction of the BLT in our institution

From January 2014 to December 2015, 622 patients receiving EAT for suspected sepsis were screened for eligibility, among whom 154 patients had ≥1 *Enterobacteriaceae* strain isolated in culture; 61 of these patients were finally included as cases and were analysed in the BLT-guided adaptation group (Fig. 2). To recruit controls in the comparative conventional adaptation group, 671 patients receiving EAT from January 2012 to December 2013 were screened for eligibility, among whom 139 had ≥1 *Enterobacteriaceae* strain isolated in culture; 61 of these patients were finally included and analysed. Baseline characteristics of the patients included were similar in the two groups (Table 1).

**Table 1:**
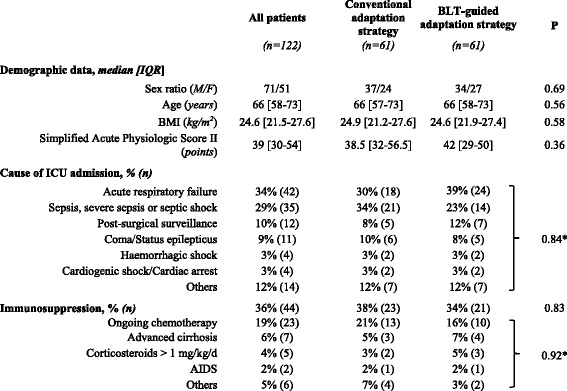
Baseline characteristics of patients

*BLT* BetaLACTA® test, *M/F* male/female, *BMI* body mass index. *Fisher exact test was used due to multiple categorical variables

### Infections

Characteristics of infections and EAT were similar in the two groups (Table 2). Pneumonia was the main source of infection. Fifty-six percent of patients had severe sepsis or septic shock. *E. coli* was the main *Enterobacteriaceae* isolated in culture, while inducible-AmpC *Enterobacteriaceae* were isolated in 24% of patients. Fifty-eight percent of *Enterobacteriaceae* were wild-type, while ESBL-producing *Enterobacteriaceae* were isolated in 20%.

**Table 2:**
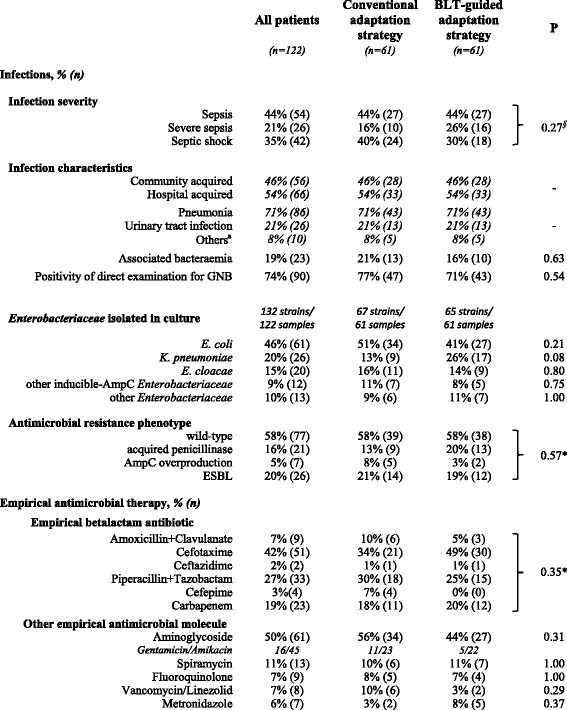
Characteristics of infections and antimicrobial therapies at inclusion

*BLT* BetaLACTA® test, *GNB* Gram-negative bacilli, *ESBL* Extended-spectrum beta-lactamases.

^a^Peritonitis (n = 3), pleurisy (n = 1) and soft-tissue abscess (n = 1)

*Fisher exact test or ^§^Chi^2^ test used due to multiple categorical variables

### Antimicrobial therapies

Patients were empirically treated with cefotaxime (42%), piperacillin/tazobactam (27%) and carbapenems (19%), without differences between groups (Table 2). Fifty percent of patients received an aminoglycoside as empirical dual antimicrobial therapy.

**Fig. 3:**
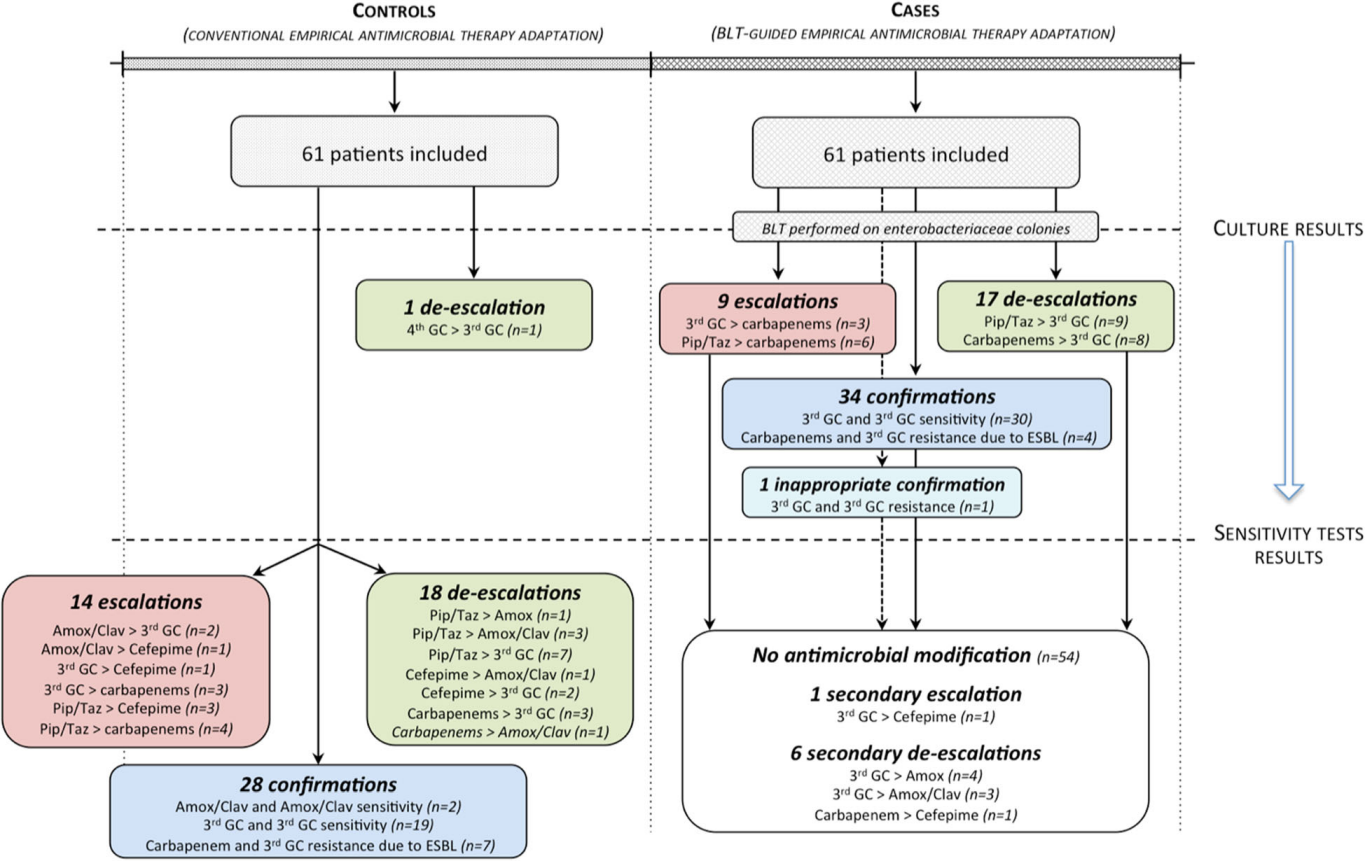
Empirical antimicrobial therapy adaptations in the conventional and BetaLACTA® test (*BLT*)-guided groups. In the conventional adaptation group, only one early de-escalation occurred once the results of the culture of the microbiological sample were available. The other 32 modifications occurred after the results of the antibiotic susceptibility tests, in particular 14 escalations for which patients received an inappropriate beta-lactam antibiotic. Conversely, in the BLT-guided adaptation group, 9 early escalations and 17 early de-escalations occurred once the results of the culture of the microbiological samples were available, among which no false positives and one false negative occurred. *Amox.* amoxicillin, *Clav.* clavulanate, *Pip.* piperacillin, *Taz.* Tazobactam, *ESBL* Extended-spectrum beta-lactamases, ^*3rd*^ *GC* and ^*4th*^ *GC*, 3^rd^ and 4^th^ generation cephalosporins

Eighty-four percent (51/61) and 77% (47/61) of cases and controls received an appropriate empirical beta-lactam respectively (*p* = 0.45) (Table 3 and Fig. 3). Once the results of the culture of the microbiological sample were available, no escalation of the inappropriate empirical beta-lactam occurred in the conventional adaptation group, whereas nine escalations were made in the BLT-guided adaptation group, reaching 98% of appropriateness (*p* < 0.01 compared to controls (see Fig. 3 and Additional file 3: Table S1 for individual descriptions). One false negative occurred in the BLT-guided adaptation group, for a healthcare-associated urinary tract infection due to acquired cephalosporinase-overproducing *E. coli*, empirically treated with Cefotaxime and definitively escalated to Cefepime according to the AST result after 64 hours. BLT-guided escalations occurred from 3GC (n = 3) and piperacillin-tazobactam (*n* = 6) to carbapenems. These nine cases were retrospectively reviewed and empirical beta-lactam complied to practice guidelines in all cases. The time between initiation of the inappropriate antimicrobial therapy and its escalation was 50.5 [48-73] hours in controls, significantly reduced to 27 [24–28] hours in cases with BLT-guided antimicrobial adaptation (*p* < 0.01) (Table 3).

**Table 3:** Endpoints and outcomes

	All patients	Conventional adaptation strategy	BLT-guided adaptation strategy	*p*
	(n = 122)	(n = 61)	(n = 61)	
Appropriate empirical antimicrobial therapy, % (*n*)	80% (98)	77% (47)	84% (51)	0.45
Early escalation, % (*n*)	7% (9)	0% (0)	15% (9)	<0.01*
Appropriate antimicrobial therapy after culture results, % (*n*)	88% (107)	77% (47)	98% (60)	<0.01
Delay before antimicrobial therapy escalation (h)	48 (28–60)	50.5 (48–73)	27 (24–28)	<0.01^#^
Optimal empirical antimicrobial therapy, % (*n*)	51% (62)	46% (28)	56% (34)	0.33
Early adaptation, % (*n*)	22% (27)	2% (1)	43% (26)	<0.01*
escalation	7% (9)	0% (0)	15% (9)	
de-escalation	15% (18)	2% (1)	28% (17)	
Optimal antimicrobial therapy after culture results, % (*n*)	71% (87)	48% (29)	95% (58)	<0.01
Delay before antimicrobial therapy adaptation (h)	49 (31.5–65)	58 (48–72)	28 (24.5–47)	<0.01^#^
Time to apyrexia (h)	24 (24–72)	24 (24–48)	30 (24–72)	0.19^#^
Time to leukocytosis <10.000/mm^3^ (days)	6 (3–13)	5 (3–12)	6 (3–15)	0.37^#^
Time to mechanical weaning (days)	5 (3–9)	4.5 (3–9)	5 (4–8)	0.51^#^
Length of ICU stay (days)	10 (5–16)	11 (6–17)	10 (5–15)	0.48
Mortality, % (*n*)	15% (18)	16% (10)	13% (8)	0.80

*BLT* BetaLACTA® test

*Fisher exact test was used due to multiple categorical variables

^#^Wilcoxon test (non-paired) was used due to data not present for patients without antimicrobial escalation/adaptation, or for patients who had never been febrile, had hyperleukocytosis, nor been ventilated

Forty-six percent (28/61) and 56% (34/61) of patients received an optimal empirical beta-lactam in the conventional and BLT-guided adaptation group respectively (*p* = 0.33; Table 3). Once the results of the culture of the microbiological sample were available, one adaptation occurred in the conventional adaptation group, while 26 adaptations were made in the BLT-guided adaptation group (43% of patients, 9 escalations and 17 de-escalations), reaching 95% of the optimum (*p* < 0.01 compared to the conventional group (Fig. 3 and Additional file 3: Table S1)). De-escalations occurred from carbapenems (n = 8) and piperacillin-tazobactam (n = 9) to cefotaxime. These 17 cases were retrospectively reviewed and the empirical beta-lactam had complied to practice guidelines in all but one case (piperacillin-tazobactam prescription that should have been carbapenem). The time between initiation of the non-optimal antimicrobial therapy and its adaptation was 58 (48–72) hours in controls, significantly reduced to 28 (24.5–47) hours in cases with BLT-guided antimicrobial adaptation (*p* < 0.01). BLT-guided early adaptations occurred more frequently in healthcare-associated than in community-acquired infections (18/33 vs. 8/28; *p* = 0.04).

### Variables associated with appropriate and optimal antimicrobial therapies

We performed multivariate analysis to determine the factors associated with appropriate and optimal antimicrobial therapies once the results of the culture of the microbiological sample were available. The following variables were entered into the model: age, Simplified Acute Physiology Score (SAPS) II severity score, presence of immunosuppression, recent travel in an ESBL-endemic region, ICU length of stay before inclusion, presence of associated bacteraemia, concomitant septic shock (vs. sepsis/severe sepsis), healthcare-associated infection (vs. community-acquired), pneumonia (vs. other sites of infection), positivity of microscopic Gram-staining examination for GNB, and use of the BLT to adapt antimicrobial therapy. In the final model, the presence of associated bacteraemia was associated with a lower rate of appropriate antimicrobial therapy (OR = 0.28 (0.08–1.01), *p* = 0.048), while BLT use was the only independent factor associated with a higher rate of appropriate antimicrobial therapy (OR = 18 (3.4–333.8), *p* = 0.006) (Additional file 4: Table S2). Similarly, use of the BLT was the only independent factor associated with a higher rate of optimal antimicrobial therapy (OR = 35.5 (9.6–231.9), *p* < 0.001) (Additional file 4: Table S3).

### Safety and outcomes

No false positive results of the BLT and one false negative result (leading to a delayed escalation) occurred. Time to reach apyrexia or normal leukocytosis was similar in the two groups among patients with fever or hyperleulocytosis at diagnosis (Table 3). Length of mechanical ventilation in patients with respiratory failure, ICU length of stay and mortality were similar in the two groups (Table 3).

## Discussion

Our main findings can be summarized as follows: in our study, the BLT-guided strategy led to: (1) a higher rate of appropriate antimicrobial therapy being administered due to early escalations of empirical beta-lactam prescriptions and (2) a higher rate of optimal antimicrobial therapy being administered due to early de-escalations of beta-lactam prescriptions that were too broad-spectrum.

One of the most challenging problems in clinical practice is that despite the use of updated guidelines, about 15% of ICU patients receive inappropriate EAT [3, 9–11]. We observed a similar rate of patients receiving inappropriate EAT in this study. This is of particular concern because inappropriate EAT leads to higher mortality in the ICU [2]. However, an across-the-board prescription of empirical carbapenems in the most severely ill patients is not acceptable, as the link between carbapenem consumption and the emergence of carbapenemase-producing *Enterobacteriaceae* or multi-drug resistant non-fermenting GNB is by now well-demonstrated [21–25], even with a short exposure [12]. Thus, the World Health Organization [13] and national plans for controlling antibiotic consumption [26] have encouraged the development of rapid diagnostic tests evaluating bacterial resistance, which should ideally enable both early escalation in the case of inappropriate therapy and early de-escalation in the case of empirical therapy that is too broad-spectrum. To our knowledge, no test meeting these requirements has yet been validated for GNB in a clinical setting. Taking into account the very good diagnostic performance of the BLT [14–17, 27], we performed this pilot study and demonstrated that the BLT is a promising approach to reach this dual objective. Indeed, the BLT-guided adaptation of antimicrobial therapy independently increased its appropriateness and optimality to 98% and 95%, respectively, within a median of 30 hours before the availability of antibiotic susceptibility test results. Theoretically, this could have additive beneficial effect. First, by decreasing the duration of inappropriate beta-lactam therapy, the use of the BLT may be associated with improvement in infection cure. Second, by decreasing the patient’s exposure to broad-spectrum beta-lactams, the use of the BLT may be associated with better preservation of intestinal microbiota and lower acquisition of multi-resistant bacteria carriage [12], which have been suggested to contribute to better outcome [20]. Due to the incidence of these events, demonstration of a BLT-related improvement in outcome would need additional studies including larger samples, especially as mortality may not be influenced by inappropriate EAT in the less severely ill patients [28] or in some low-risk sites of infection [29] in which definite antimicrobial therapy is appropriate.

As for other diagnostic tests in the field of infectious diseases, clinical uptake of the BLT should be based on multifaceted evaluation [30]. Although several studies have previously reported the very good intrinsic diagnostic performance of the BLT [14–17, 27], we reported its added clinical value for the first time, even when empirical beta-lactam antibiotic was chosen according to guidelines. The cost of the procedure also appears as strength since the device is inexpensive (3.5 €) and takes little time to perform (2 minutes for the assay and 15 minutes for the incubation prior to analyze the result). When compared with other biological tests, the BLT appears cost-effective. Indeed, its cost is quite similar to that of the disk-diffusion AST (3 €), and it is much cheaper than molecular detection of antibiotic resistance, such as GeneXpert® methicillin-resistant *Staphylococcus aureus* detection (18 €). To further illustrate the size of the required investment, in our institution the cost of BLT is amortized after the first and third doses of cefotaxime when de-escalating from carbapenems and piperacillin-tazobactam, respectively (Additional file 5: Figure S1).

Our study may have several limitations. First, the number of patients included in each group may appear relatively small. However, according to the sample size calculation performed prior to the beginning of the study, the numbers were appropriate to demonstrate the superiority of the BLT-guided adaptation strategy in improving the appropriateness and optimality of the antimicrobial therapy once the results of microbiological sample culture are available. We did not observe differences in morbidity and mortality between patients treated according to conventional and BLT-guided strategies, but our study is likely to be underpowered to detect small differences in the incidence of safety parameters on the one hand, and improvement in the outcome of patients with early beta-lactam escalation on the other hand. Second, although the sensitivity of the BLT is above 95% for ESBL detection, lower sensitivity (around 60%) has been reported for detection of AmpC-overproducing *Enterobacteriaceae* [14, 15]. Indeed, we reported one false negative for a cephalosporinase-overproducing GNB, which led to delayed escalation to Cefepime, similar to what would have occurred without the use of the BLT. Nevertheless, our study demonstrated that this event is much less incidental in clinical practice than the BLT-guided early escalations of inappropriate EAT, suggesting a BLT benefit/risk balance in favour of its use. Finally, although our case-control study is the first to report the potential utility of a rapid phenotypic test detecting GNB resistance to 3GC in clinical settings, definitive evidence will require a large, randomized, controlled trial.

Looking ahead, if the use of the BLT for *Enterobacteriaceae* colonies saves time in assessing the adequacy of antimicrobial therapy compared to the AST, recent developments could lead to another 24 hours being saved. Indeed, several protocols enable the efficient detection of *Enterobacteriaceae* that produce ESBL directly from the microbiological samples [17, 27, 31]. As such, it could be hypothesized that the direct use of the BLT on a microbiological sample that is positive for GNB on direct examination could shorten the duration of inappropriate EAT to a couple of hours.

## Conclusion

Despite the use of updated guidelines and a good compliance rating from physicians, one out of every six ICU patients receive inappropriate EAT. In more cases, empirical antimicrobial therapy is too broad-spectrum, which negatively impacts patients’ and hospital’s bacterial ecologies. The BLT is a simple, accurate, inexpensive susceptibility test, which is promising for early beta-lactam adaptation from the first 24 hours following the beginning of sepsis management. A randomized, controlled trial is needed to definitely demonstrate the impact of a BLT-guided strategy on the patient’s outcome.

## Additional files

Additional file 1: additional methods. (DOCX 40 kb)

Additional file 2: institutional protocols for selection of the empirical antimicrobial therapy in case of pneumonia, pyelonephritis and peritonitis. (PDF 54 kb)

Additional file 3: Table S1. Individual early BLT-guided beta-lactam adaptations and confirmations. (PDF 59 kb)

Additional file 4: Table S2. (*Predictors of an appropriate antimicrobial therapy*) and **Table S3**. (*Predictors of an optimal antimicrobial therapy*). (DOCX 21 kb)

Additional file 5: Figure S1. Comparison of the antimicrobial therapy-related costs between the conventional strategy (*left*) and the BLT-guided strategy (*right*) for de-escalation from piperacillin-tazobactam (*first line*), imipenem (*middle line*) and meropenem (*last line*) to cefotaxime. Given antimicrobial prices in our structure and considering antimicrobial administration three times a day, BLT-guided de-escalation from piperacillin-tazobactam to cefotaxime costs the same as a 48-hour empirical administration of piperacillin-tazobactam, while BLT-guided de-escalation from carbapenem to cefotaxime is cheaper than a 48-hour empirical administration of imipenem (-4€) or meropenem (-10€). (PNG 160 kb)

## Abbreviations

3GC: 3^rd^ Generation cephalosporins
AST: Antibiotic susceptibility test
BLT: BetaLACTA® test
EAT: Empirical antimicrobial therapy
ESBL: Extended-spectrum beta-lactamases
GNB: Gram-negative bacilli
ICU: Intensive care unit
MDR: Multi-drug resistant

## References

[1] Dombrovskiy VY, Martin AA, Sunderram J, Paz HL (2007). Rapid increase in hospitalization and mortality rates for severe sepsis in the United States: a trend analysis from 1993 to 2003. Crit Care Med.

[2] Kumar A, Ellis P, Arabi Y (2009). Initiation of inappropriate antimicrobial therapy results in a fivefold reduction of survival in human septic shock. Chest.

[3] Zahar J-R, Timsit J-F, Garrouste-Orgeas M (2011). Outcomes in severe sepsis and patients with septic shock: pathogen species and infection sites are not associated with mortality. Crit Care Med.

[4] InVS/REA-Raisin. Surveillance de la consommation des antibiotiques (Antimicrobials consumption monitoring) (2011) [Internet]. 2013. Available from: http://www.invs.sante.fr/Publications-et-outils/Rapports-et-syntheses/Maladies-infectieuses/2013/Surveillance-de-la-consommation-des-antibiotiques-Reseau-ATB-Raisin-Resultats-2011. 14 June 2017.

[5] European Centre for Disease Prevention and Control. Antimicrobial resistance interactive database (2013) [Internet]. 2013. Available from: http://www.ecdc.europa.eu/en/healthtopics/antimicrobial_resistance/database/Pages/database.aspx. 15 Apr 2017.

[6] Bretonnière C, Leone M, Milési C (2015). Strategies to reduce curative antibiotic therapy in intensive care units (adult and paediatric). Intensive Care Med.

[7] Dellinger RP, Levy MM, Rhodes A (2013). Surviving Sepsis Campaign: international guidelines for management of severe sepsis and septic shock, 2012. Intensive Care Med.

[8] Leone M, Bechis C, Baumstarck K (2014). De-escalation versus continuation of empirical antimicrobial treatment in severe sepsis: a multicenter non-blinded randomized noninferiority trial. Intensive Care Med.

[9] Garnacho-Montero J, Garcia-Garmendia JL, Barrero-Almodovar A, Jimenez-Jimenez FJ, Perez-Paredes C, Ortiz-Leyba C (2003). Impact of adequate empirical antibiotic therapy on the outcome of patients admitted to the intensive care unit with sepsis. Crit Care Med.

[10] Leone M, Bourgoin A, Cambon S, Dubuc M, Albanèse J, Martin C (2003). Empirical antimicrobial therapy of septic shock patients: adequacy and impact on the outcome. Crit Care Med.

[11] Mokart D, Slehofer G, Lambert J (2014). De-escalation of antimicrobial treatment in neutropenic patients with severe sepsis: results from an observational study. Intensive Care Med.

[12] Armand-Lefèvre L, Angebault C, Barbier F (2013). Emergence of imipenem-resistant gram-negative bacilli in intestinal flora of intensive care patients. Antimicrob Agents Chemother.

[13] Organisation Mondiale de la Santé/World Health Organization. Projet de plan mondial pour combattre la résistance aux antimicrobiens (Draft global action plan on antimicrobial resistance) [Internet]. 2014. Available from: http://apps.who.int/gb/ebwha/pdf_files/EB136/B136_20-fr.pdf. 14 June 2017.

[14] Renvoisé A, Decré D, Amarsy-Guerle R (2013). Evaluation of the βLacta test, a rapid test detecting resistance to third-generation cephalosporins in clinical strains of Enterobacteriaceae. J Clin Microbiol.

[15] Morosini MI, García-Castillo M, Tato M (2014). Rapid detection of β-lactamase-hydrolyzing extended-spectrum cephalosporins in Enterobacteriaceae by use of the new chromogenic βLacta test. J Clin Microbiol.

[16] Compain F, Bensekhri H, Rostane H, Mainardi J-L, Lavollay M (2015). β LACTA test for rapid detection of Enterobacteriaceae resistant to third-generation cephalosporins from positive blood cultures using briefly incubated solid medium cultures. J Med Microbiol.

[17] Gallah S, Decré D, Genel N, Arlet G (2014). The β-Lacta test for direct detection of extended-spectrum-β-lactamase-producing Enterobacteriaceae in urine. J Clin Microbiol.

[18] von Elm E, Altman DG, Egger M (2008). The Strengthening the Reporting of Observational Studies in Epidemiology (STROBE) statement: guidelines for reporting observational studies. J Clin Epidemiol.

[19] Weiss E, Zahar J-R, Lesprit P (2015). Elaboration of a consensual definition of de-escalation allowing a ranking of β-lactams. Clin Microbiol Infect.

[20] Garnacho-Montero J, Gutiérrez-Pizarraya A, Escoresca-Ortega A (2014). De-escalation of empirical therapy is associated with lower mortality in patients with severe sepsis and septic shock. Intensive Care Med.

[21] Ogutlu A, Guclu E, Karabay O, Utku AC, Tuna N, Yahyaoglu M (2014). Effects of Carbapenem consumption on the prevalence of Acinetobacter infection in intensive care unit patients. Ann Clin Microbiol Antimicrob.

[22] Peña C, Guzmán A, Suarez C (2007). Effects of carbapenem exposure on the risk for digestive tract carriage of intensive care unit-endemic carbapenem-resistant Pseudomonas aeruginosa strains in critically ill patients. Antimicrob Agents Chemother.

[23] McLaughlin M, Advincula MR, Malczynski M, Qi C, Bolon M, Scheetz MH (2013). Correlations of antibiotic use and carbapenem resistance in enterobacteriaceae. Antimicrob Agents Chemother.

[24] Mantzarlis K, Makris D, Manoulakas E, Karvouniaris M, Zakynthinos E (2013). Risk factors for the first episode of Klebsiella pneumoniae resistant to carbapenems infection in critically ill patients: a prospective study. BioMed Res Int.

[25] Routsi C, Pratikaki M, Platsouka E (2013). Risk factors for carbapenem-resistant Gram-negative bacteremia in intensive care unit patients. Intensive Care Med.

[26] Ministère français de la Santé (French Health Ministry). Plan national d’alerte sur les antibiotiques (National plan on antimicrobials) - 2011/2016 [Internet]. 2011. Available from: http://www.sante.gouv.fr/IMG/pdf/plan_antibiotiques_2011-2016_DEFINITIF.pdf. 14 June 2017.

[27] Walewski V, Podglajen I, Lefeuvre P (2015). Early detection with the β-LACTA™ test of extended-spectrum β-lactamase-producing Enterobacteriaceae in blood cultures. Diagn Microbiol Infect Dis.

[28] Lee C-C, Chang C-M, Hong M-Y, Hsu H-C, Ko W-C (2013). Different impact of the appropriateness of empirical antibiotics for bacteremia among younger adults and the elderly in the ED. Am J Emerg Med.

[29] Joo E-J, Kang C-I, Ha YE (2011). Impact of inappropriate empiric antimicrobial therapy on outcome in Pseudomonas aeruginosa bacteraemia: a stratified analysis according to sites of infection. Infection.

[30] Banoo S, Bell D, Diagnostics Evaluation Expert Panel TDR (2010). Evaluation of diagnostic tests for infectious diseases: general principles. Nat Rev Microbiol.

[31] Gallah S, Benzerara Y, Garnier M (2015). Evaluation of the chromogenic betaLACTA™ test for early detection of ESBL-producing enterobacteriacae directly on respiratory samples - Poster cession of the 35th interdisciplinary meeting on anti-infectious chemotherapy (RICAI) - 2015.

