# Supplementary material for: Evaluation of early antimicrobial therapy adaptation guided by the BetaLACTA® test: a case-control study
Source: Crit Care. 2017 Jun 28;21:161. doi: 10.1186/s13054-017-1746-6 (PMC5488410; doi:10.1186/s13054-017-1746-6)
Supplement: Supplementary file 1 — additional methods. (DOCX 40 kb) [file 13054_2017_1746_MOESM1_ESM.docx]

**BetaLACTA^®^ test Use for Early Improvement of Antimicrobial Therapy Adequacy in Intensive Care Unit Patients with Infections caused by *Enterobacteriaceae*: a Pre-Post Quasi-Experimental Study**

**Supplemental Methods**

**Ethical considerations**

This case-control study was conducted following the STROBE statements (*see STROBE checklist available as an additional file*) and approved by the Institutional Review Board (Comité de Protection des Personnes “Ile de France VI”, Paris, France, ID-RCB 2015-A00169-40), and by the French national commission on digital storage of personal data (CNIL, n°1832114). Due to routine introduction of a CE marked test in our lab, patient approval was waived by the ethics committee. Consequently, information was given to patients or next-of-kin who could decline inclusion in the study.

**Patients and Empirical Antimicrobial Therapy**

After BLT introduction as a routine susceptibility test in our lab, all ICU patients were screened daily for eligibility. Eligibility criteria were the presence of an infection caused by at least one *Enterobacteriaceae* species and requiring an EAT. Infection was diagnosed on the presence of: 1) at least two criteria of the Systemic Inflammatory Response Syndrome, 2) symptoms suggesting organ infection (such as purulent trachea-bronchial secretions, hypoxemia or chest infiltration for pneumonia for instance), and 3) ≥ 1 bacterial species (including at least one *Enterobacteriaceae* species) at concentration upon the usual positivity threshold isolated from the microbiological sample culturing (such as ≥10^5^ cfu/mL for catheter-associated urinary tract infection for instance).

Exclusion criteria were: 1) antimicrobial therapy withdrawal within the first 48 hours of treatment due to diagnosis reclassification from infection to colonization, 2) existence of a concomitant infection preventing antimicrobial de-escalation, 3) moribund patient or patient in whom a procedure of withdrawing life-sustaining treatment was decided.

Consecutive eligible patients were prospectively included as “cases” in the “BLT group”. One “control” (i.e. patient with AST-guided EAT adaptation), matched both on the site and the community-acquired or healthcare-associated status of the infection, was recruited per case among the last patients who presented an *Enterobacteriaceae* infection before BLT introduction, defined using the same definition criteria than for the cases of the BLT group.

Composition of the EAT was prescribed according to local practice guidelines adapted from international guidelines in collaboration with the microbiology and infectious disease departments staff members. Local prescription guidelines for community-acquired and hospital acquired pneumonia, urinary tract infection and peritonitis are provided at the end of this document. Briefly, cefotaxime was mainly selected for community-acquired and early-onset nosocomial infections. Piperacillin-tazobactam, cefepime, imipenem-cilastatin, doripenem or meropenem were selected for late-onset nosocomial infections. An aminoglycoside was added in case of septic shock or of high suspicion of *Pseudomonas aeruginosa*.

Senior intensivists discussed all antibiotic prescriptions within the 24 hours following their initiation. Moreover, all carbapenem prescriptions were discussed once a week with referents from the infectious disease and microbiology departments. ICU physicians and local empirical prescription guidelines did not change during the two study periods, except for withdrawal of doripenem from August 2014.

**Microbiological procedures**

No significant change in microbiological practice occurred during the two study periods. After microbiological validation of sample culturing, a BLT was performed by a trained microbiologist on *Enterobacteriaceae* colonies as recommended by the manufacturer. Briefly, freshly isolated *Enterobacteriaceae* colonies obtained after 16 to 24h of culture of the microbiological sample were picked up until a 1 µl loop was full, and then put into a micro-tube containing 1 drop of BLT reagent R1 and 1 drop of BLT reagent R2. Then, the micro-tube was left for 15 minutes at room temperature, before reading the result. BLT was interpreted as positive if colour turned to red, purple or orange, and negative if colour remained yellow. Device cost was 3.5€.

AST was performed by the disk diffusion method the same day as the BLT. If several *Enterobacteriaceae* were isolated in culture, BLT and AST were performed on each species, and EAT was adapted considering the most resistant phenotype.

**Antimicrobial adaptation strategies**

In the “conventional adaptation group” (i.e. the “controls”), empirical beta-lactam could be modified if needed on results of microbiological sample culturing, notably *Enterobacteriaceae* identification, and was then definitively adapted to AST.

In the “BLT-guided adaptation group” (i.e. the “cases”), empirical beta-lactam was adapted to culture and BLT results as shown in ***Figure 1***. When needed, attending physician could secondarily adapt the BLT-guided beta-lactam to the results of AST.

**Definitions and End Points**

An “appropriate antimicrobial therapy” was defined as use of a beta-lactam active on the identified *Enterobacteriaceae* as determined by AST as gold standard.

An “optimal antimicrobial therapy” was defined as use of a beta-lactam belonging to a class with a narrow spectrum among all the classes of beta-lactams active on the identified *Enterobacteriaceae,* as defined by Weiss and colleagues ^18^ : amoxicillin, amoxicillin/clavulanate or cefotaxime for wild-type *Enterobacteriaceae;* cefepime for AmpC-overproducing *Enterobacteriaceae*; and carbapenem for ESBL-producing *Enterobacteriaceae*.

As BLT is not aimed to shorten the time between sampling and pathogen identification, but between pathogen identification and determination of its antibiotic susceptibility, the primary aim of the study was to determine if the BLT-guided adaptation increased the proportion of appropriate antimicrobial therapy once the results of the culture of the microbiological sampling were available, compared to conventional adaptation. The secondary aim of the study was to determine if the BLT-guided adaptation increased the proportion of optimal antimicrobial therapy at the same time point.

**Statistical analysis**

The sample size estimation was based on a Chi-square test for comparison of two proportions. Assuming a proportion of 80% of appropriateness of the EAT in the conventional adaptation group ^9–11 20^, 60 patients per group were needed to show an increase to a proportion of 95% of appropriateness of the antimicrobial therapy in the BLT-guided adaptation group, with an alpha risk of 5 % and a power of 80 %.

Differences in categorical variables were analyzed using the chi-square or the Fisher exact test or the McNemar test for matched patients, as appropriate. Continuous variables were reported as median [interquartile range], and were compared using the matched-paired Wilcoxon rank-sum test. When some data were missing due to the absence of the considered parameter in at least one patient of each pair (such as absence of fever, absence of antimicrobial adaptation due to optimal empirical treatment, etc.), the non-paired Wilcoxon test was used.

Further, multivariate analyses were performed to determine variables independently associated with appropriate and optimal antimicrobial therapies. Both covariates known to impact the choice of the EAT determined *a priori* and those with P<0.20 in bivariate analyses were entered into multivariate regression models with variable selection based on a stepwise backward and forward elimination procedure using P values.

All included patients were considered for analysis. P<0.05 was considered as significant. Statistical analysis was performed with GraphPad Prism 6 (GraphPad Software, San Diego, CA, USA) and R3.3.2 (http://www.R-project.org).
