# Supplementary material for: Evaluation of early antimicrobial therapy adaptation guided by the BetaLACTA® test: a case-control study
Source: Crit Care. 2017 Jun 28;21:161. doi: 10.1186/s13054-017-1746-6 (PMC5488410; doi:10.1186/s13054-017-1746-6)
Supplement: Supplementary file 2 — institutional protocols for selection of the empirical antimicrobial therapy in case of pneumonia, pyelonephritis and peritonitis. (PDF 54 kb) [file 13054_2017_1746_MOESM2_ESM.pdf]

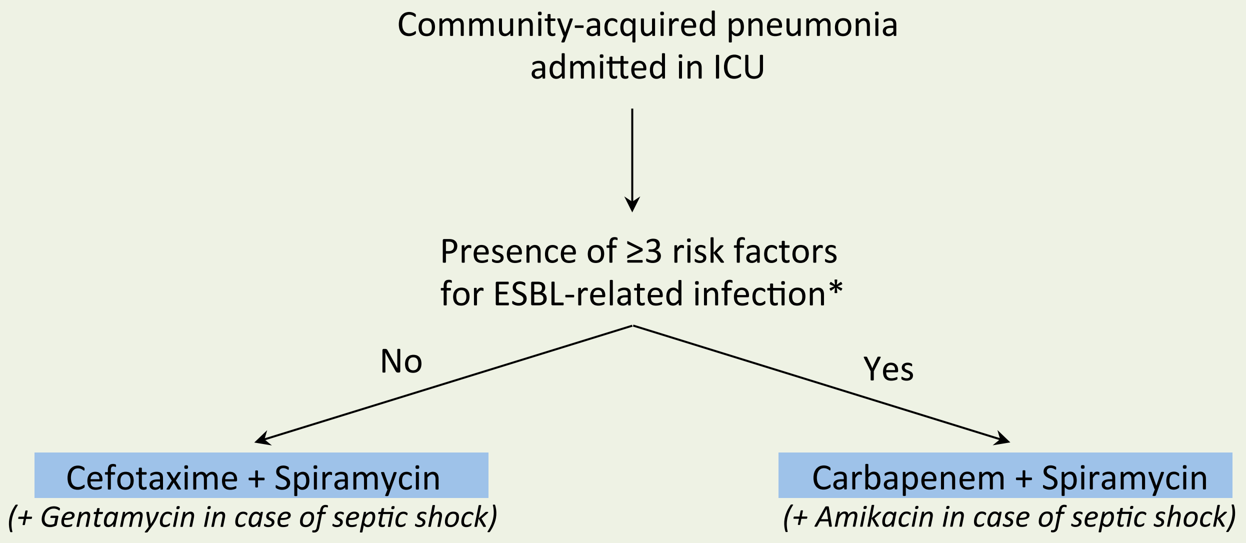

* *Antimicrobial therapy (amoxicillin/clavulanate, 3rd generation cephalosporins or fluoroquinolons) in the last 3 months, hospital admission for more than 48h in the last 3 months, hospital admission in an ESBL-endemic country in the last 6 months, immunosuppression, chronic enteral nutrition, prior recurrent urinary tract disease, life in institutions or nursing home*

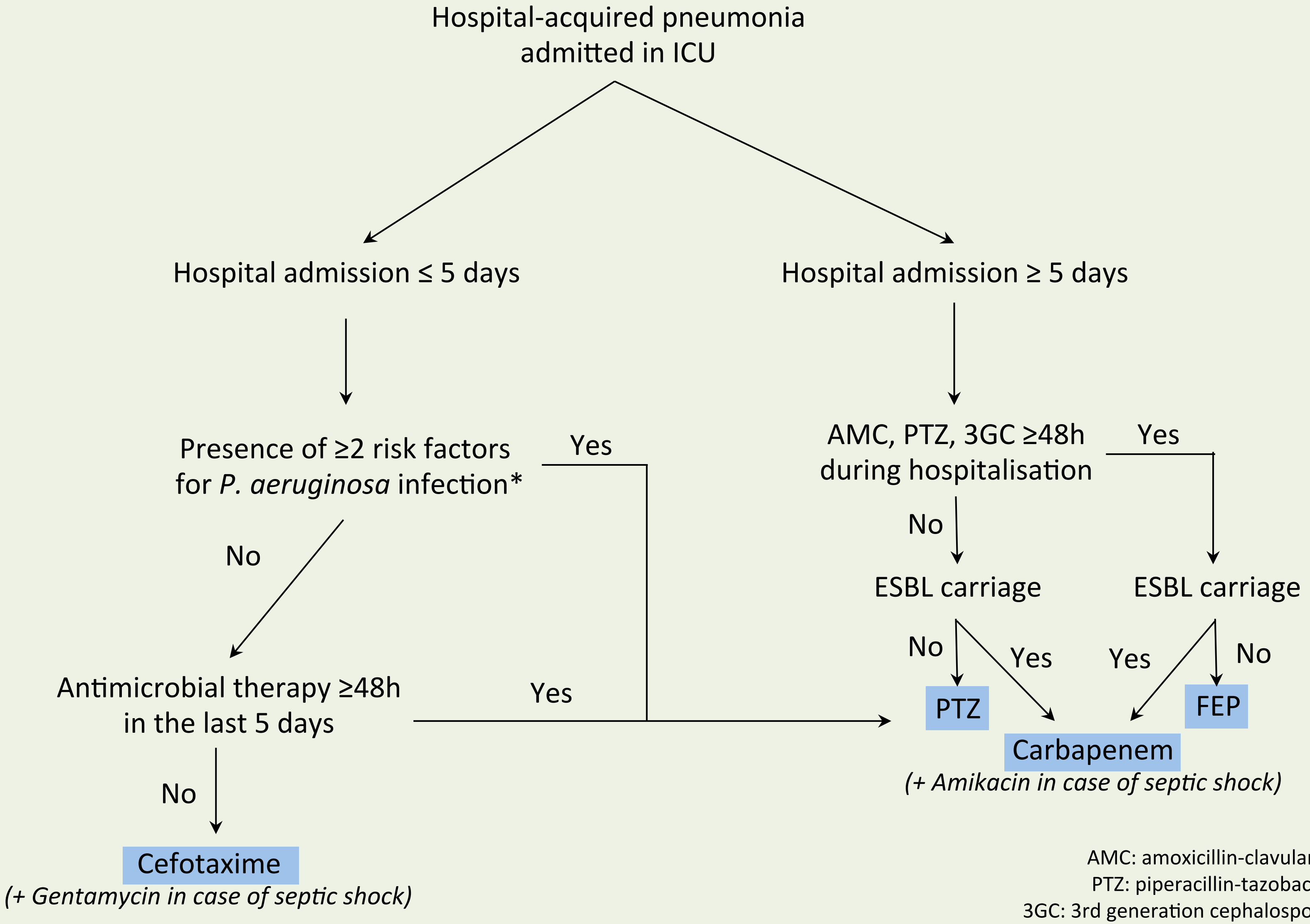

AMC: amoxicillin-clavulanate
PTZ: piperacillin-tazobactam
3GC: 3rd generation cephalosporins
FEP: cefepime

* *Antimicrobial therapy (amoxicillin/clavulanate, 3rd generation cephalosporins or fluoroquinolons) in the last 3 months or >4 times/year, hospital admission for more than 48h in the last 6 months, immunosuppression, COPD patient with FEV <30%, bronchiectasis*

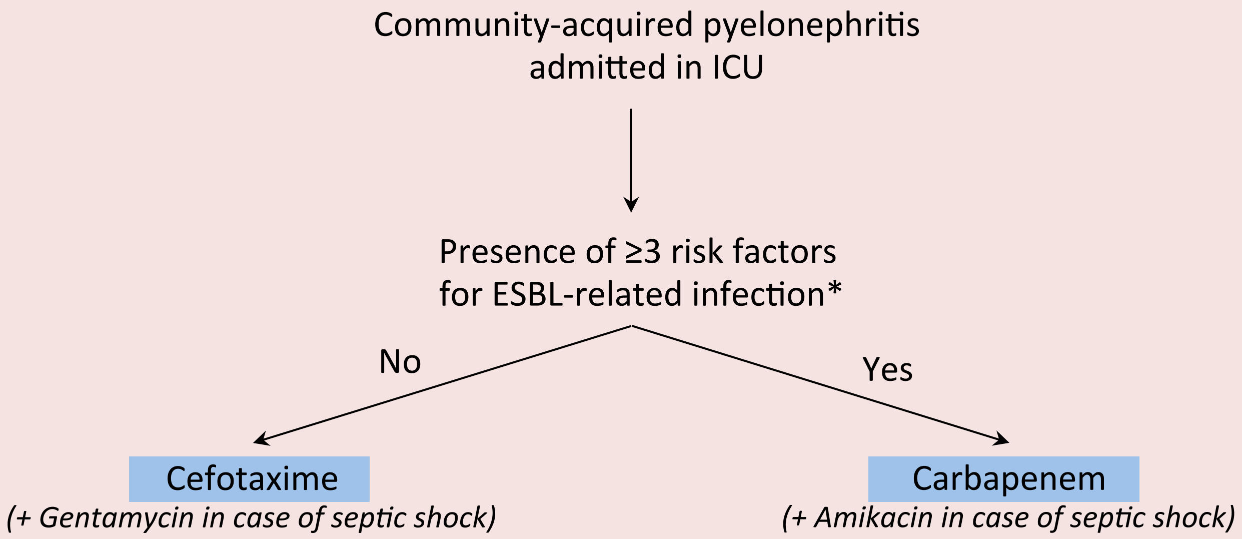

* *Antimicrobial therapy (amoxicillin/clavulanate, 3rd generatin cephalosporins or fluoroquinolons) in the last 3 months, hospital admission for more than 48h in the last 3 months, hospital admission in an ESBL-endemic country in the last 6 months, immunosuppression, chronic enteral nutrition, prior recurrent urinary tract disease, life in institutions or nursing home*

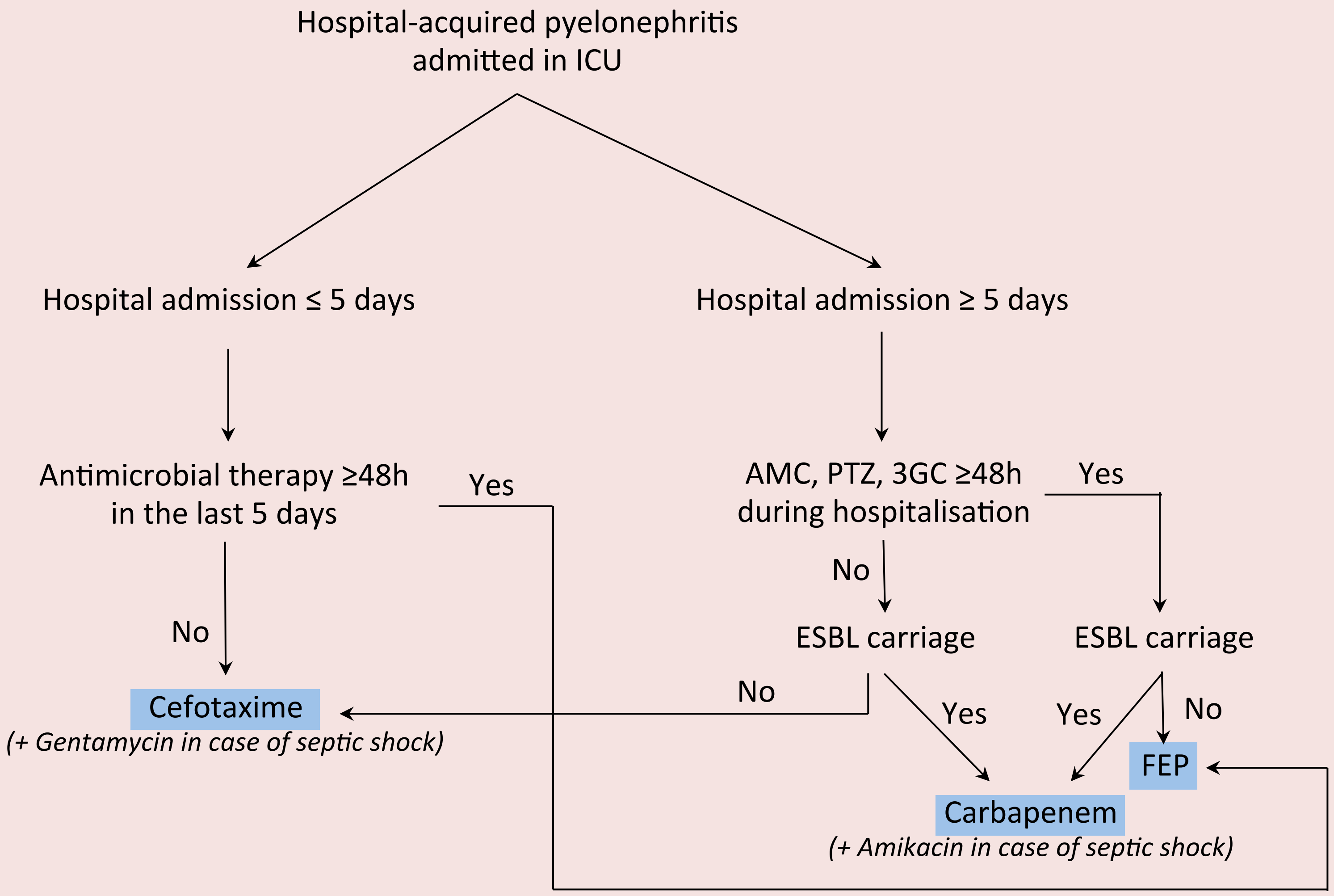

AMC: amoxicillin-clavulanate
PTZ: piperacillin-tazobactam
3GC: 3rd generation cephalosporins
FEP: cefepime

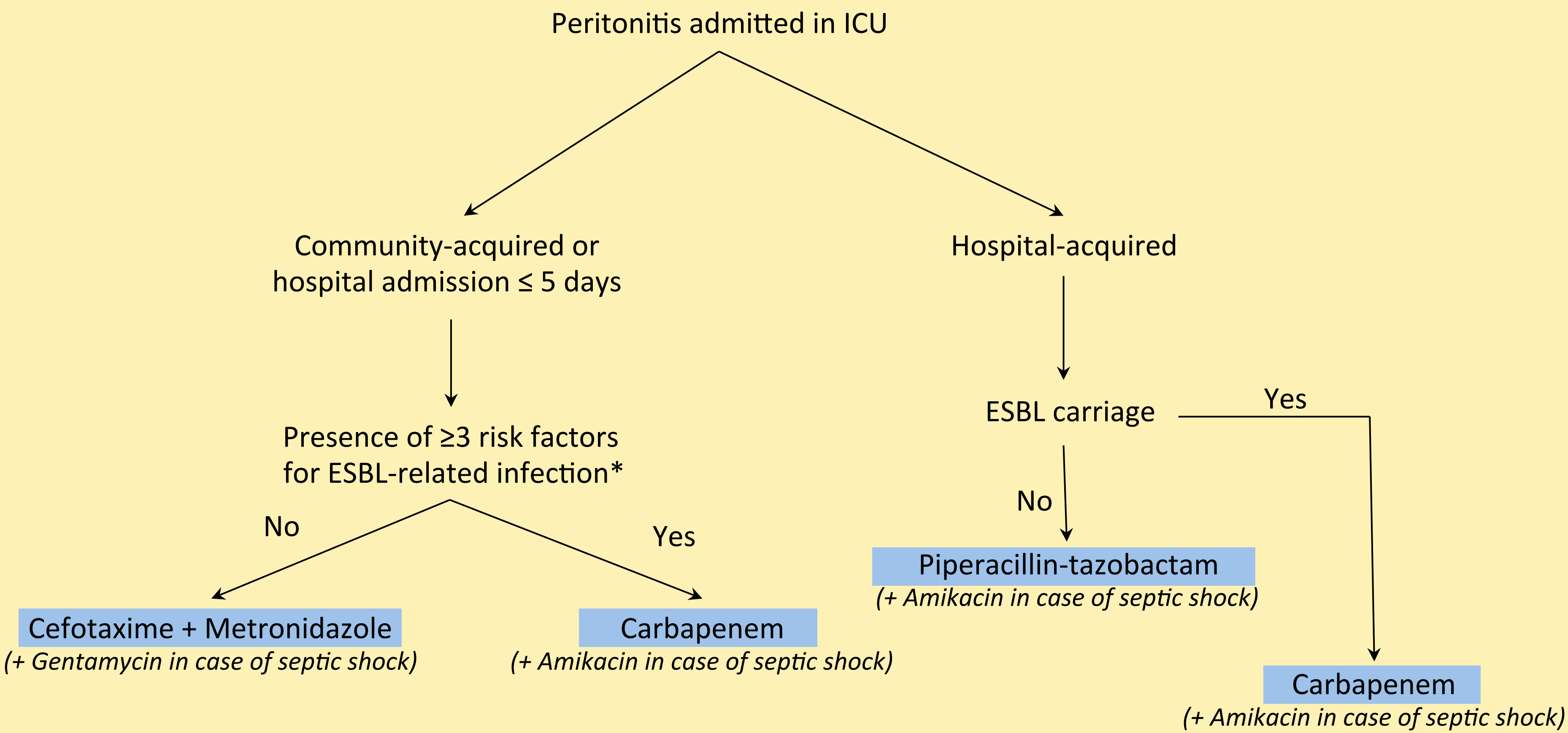

* *Antimicrobial therapy (amoxicillin/clavulanate, 3rd generatin cephalosporins or fluoroquinolons) in the last 3 months, hospital admission for more than 48h in the last 3 months, hospital admission in an ESBL-endemic country in the last 6 months, immunosuppression, chronic enteral nutrition, prior recurrent urinary tract disease, life in institutions or nursing home*
