# Supplementary material for: Evaluation of early antimicrobial therapy adaptation guided by the BetaLACTA® test: a case-control study
Source: Crit Care. 2017 Jun 28;21:161. doi: 10.1186/s13054-017-1746-6 (PMC5488410; doi:10.1186/s13054-017-1746-6)
Supplement: Supplementary file 3 — Individual early BLT-guided beta-lactam adaptations and confirmations. (PDF 59 kb) [file 13054_2017_1746_MOESM3_ESM.pdf]

Patient number	Type of infection	Site of infection	Isolated *enterobacteriaceae*	Resistance phenotype	BetaLACTA test result	Empirical antimicrobial therapy	BLT-guided adaptation	Delay: sampling > BLT result *(hours)*	Delay: sampling > antibiogram result *(hours)*	Delay: sample arrival in the lab > BLT result *(hours)*	Delay: sample arrival in the lab > antibiogram result *(hours)*
**Escalations**											
#5	Healthcare associated	Pneumonia	*Enterobacter cloacae*	AmpC overproduction	Positive	Ceftazidim	Doripenem	36	60	27	51
#8	Healthcare associated	Pneumonia	*Enterobacter cloacae*	ESBL	Positive	Cefotaxime	Doripenem	28	52	27	51
#20	Healthcare associated	Pneumonia	*Escherichia coli*	ESBL	Positive	Piperacillin + Tazobactam	Doripenem	24	52	24	52
#25	Healthcare associated	Pneumonia	*Enterobacter cloacae*	ESBL	Positive	Cefotaxime	Imipenem	28	52	28	52
#29	Healthcare associated	Pneumonia	*Escherichia coli*	ESBL	Positive	Piperacillin + Tazobactam	Meropenem	28	100	26	98
#31	Healthcare associated	Pneumonia	*Enterobacter aerogenes*	ESBL	Positive	Piperacillin + Tazobactam	Meropenem	43	67	26	50
#46	Community acquired	Pneumonia	*Klebsiella pneumoniae*	ESBL	Positive	Piperacillin + Tazobactam	Imipenem	24	72	21	69
#54	Community acquired	Pneumonia	*Escherichia coli*	ESBL	Positive	Piperacillin + Tazobactam	Meropenem	35	58	29	52
#61	Healthcare associated	UTI	*Klebsiella oxytoca*	ESBL	Positive	Piperacillin + Tazobactam	Meropenem	35	57	23	45
**De-escalations**											
#3	Healthcare associated	Pneumonia	*Enterobacter cloacae*<br>*Proteus Mirabilis*	Wild type<br>Wild type	Negative<br>Negative	Piperacillin + Tazobactam	Cefotaxime	36	60	27	51
#7	Healthcare associated	Pneumonia	*Serratia marcescens*	Wild type	Negative	Piperacillin + Tazobactam	Cefotaxime	36	60	28	52
#9	Healthcare associated	Pneumonia	*Escherichia coli*	Inhibitor-resistant TEM	Negative	Doripenem	Cefotaxime	28	52	27	51
#12	Healthcare associated	UTI	*Klebsiella pneumoniae*	Acquired penicillinase	Negative	Meropenem	Cefotaxime	27	51	26	50
#13	Healthcare associated	Pneumonia	*Citrobacter koseri*	Wild type	Negative	Piperacillin + Tazobactam	Cefotaxime	38	66	24	52
#15	Healthcare associated	Pneumonia	*Escherichia coli*	Acquired penicillinase	Negative	Piperacillin + Tazobactam	Cefotaxime	28	52	26	50
#16	Healthcare associated	UTI	*Escherichia coli*	Wild type	Negative	Imipenem	Cefotaxime	24	48	22	46
#34	Community acquired	Pneumonia	*Escherichia coli*	Acquired penicillinase	Negative	Piperacillin + Tazobactam	Cefotaxime	28	52	24	48
#35	Community acquired	Pneumonia	*Enterobacter cloacae*	Acquired penicillinase	Negative	Piperacillin + Tazobactam	Cefotaxime	40	64	27	51
#37	Healthcare associated	Pneumonia	*Enterobacter cloacae*	Wild type	Negative	Meropenem	Cefotaxime	49	73	27	51
#49	Community acquired	UTI	*Klebsiella pneumoniae*	Wild type	Negative	Meropenem	Cefotaxime	64	86	51	73
#50	Community acquired	Pneumonia	*Enterobacter cloacae*	Wild type	Negative	Piperacillin + Tazobactam	Cefotaxime	29	77	27	75
#51	Healthcare associated	Abcess	*Enterobacter cloacae*	Wild type	Negative	Imipenem	Cefotaxime	50	74	48	72
#53	Community acquired	Pneumonia	*Klebsiella pneumoniae*	Wild type	Negative	Piperacillin + Tazobactam	Cefotaxime	42	64	27	49
#55	Community acquired	UTI	*Klebsiella pneumoniae*	Wild type	Negative	Meropenem	Cefotaxime	40	63	25	48
#57	Healthcare associated	UTI	*Escherichia coli*	Acquired penicillinase	Negative	Piperacillin + Tazobactam	Cefotaxime	37	61	27	51
#59	Healthcare associated	Pneumonia	*Citrobacter koseri*	Wild type	Negative	Meropenem	Cefotaxime	23	45	22	44
**Empirical beta-lactam confirmations**											
Empirical carbapenem confirmation											
#1	Healthcare associated	Pneumonia	*Klebsiella pneumoniae*	ESBL	Positive	Doripenem	Doripenem	24	49	20	45
#41	Healthcare associated	Pneumonia	*Klebsiella pneumoniae*	ESBL	Positive	Meropenem	Meropenem	28	52	27	51
#45	Healthcare associated	Pleurisy	*Klebsiella pneumoniae*	ESBL	Positive	Imipenem	Imipenem	24	48	23	47
#60	Healthcare associated	Peritonitis	*Escherichia coli*	ESBL	Positive	Meropenem	Meropenem	59	83	47	61
Empirical Cefotaxime confirmation											
#2	Community acquired	Pneumonia	*Serratia marcescens*	Wild type	Negative	Cefotaxime	Cefotaxime	60	84	51	75
#4	Healthcare associated	Pneumonia	*Klebsiella pneumoniae*	Wild type	Negative	Cefotaxime	Cefotaxime	24	48	24	48
#6	Community acquired	Pneumonia	*Escherichia coli*	Wild type	Negative	Cefotaxime	Cefotaxime	36	60	26	50
#10	Community acquired	Pneumonia	*Klebsiella pneumoniae*	Wild type	Negative	Cefotaxime	Cefotaxime	28	52	28	52
#11	Healthcare associated	UTI	*Escherichia coli*	Acquired penicillinase	Negative	Cefotaxime	Cefotaxime	28	52	24	48
#14	Community acquired	UTI	*Escherichia coli*	Acquired penicillinase	Negative	Cefotaxime	Cefotaxime	34	62	22	50
#17	Healthcare associated	Pneumonia	*Morganella morganii*<br>*Proteus mirabilis*	Wild-type<br>Acquired penicillinase	Negative<br>Negative	Cefotaxime	Cefotaxime	24	48	24	48
#18	Community acquired	Pneumonia	*Klebsiella pneumoniae*	Wild type	Negative	Cefotaxime	Cefotaxime	28	52	24	48
#19	Healthcare associated	Pneumonia	*Escherichia coli*	Acquired penicillinase	Negative	Cefotaxime	Cefotaxime	34	58	24	48
#21	Healthcare associated	Pneumonia	*Escherichia coli*	Wild type	Negative	Cefotaxime	Cefotaxime	28	52	24	48
#22	Healthcare associated	Pneumonia	*Escherichia coli*	Wild type	Negative	Cefotaxime	Cefotaxime	44	68	28	52
#23	Healthcare associated	Pneumonia	*Klebsiella pneumoniae*	Wild type	Negative	Cefotaxime	Cefotaxime	24	52	24	52
#24	Community acquired	Pneumonia	*Escherichia coli*	Acquired penicillinase	Negative	Cefotaxime	Cefotaxime	28	52	28	52
#26	Healthcare associated	Pneumonia	*Escherichia coli*	Acquired penicillinase	Negative	Cefotaxime	Cefotaxime	28	52	28	52
#27	Community acquired	Pneumonia	*Escherichia coli*	Wild type	Negative	Cefotaxime	Cefotaxime	24	52	24	52
#28	Healthcare associated	Pneumonia	*Klebsiella pneumoniae*	Wild type	Negative	Cefotaxime	Cefotaxime	24	52	24	52
#30	Community acquired	UTI	*Escherichia coli*	Wild type	Negative	Cefotaxime	Cefotaxime	44	68	28	52
#32	Healthcare associated	Pneumonia	*Klebsiella pneumoniae*	Wild type	Negative	Cefotaxime	Cefotaxime	24	48	24	48
#33	Community acquired	UTI	*Escherichia coli*	Wild type	Negative	Cefotaxime	Cefotaxime	28	52	24	48
#36	Community acquired	Pneumonia	*Klebsiella pneumoniae*	Wild type	Negative	Cefotaxime	Cefotaxime	24	51	24	51
#38	Community acquired	Pneumonia	*Escherichia coli*	Wild type	Negative	Amoxicilin+Clavulanate	Amoxicilin+Clavulanate	43	67	27	51
#39	Community acquired	Pneumonia	*Klebsiella pneumoniae*	Wild type	Negative	Cefotaxime	Cefotaxime	43	67	27	51
#40	Community acquired	Pneumonia	*Enterobacter cloacae*	Wild type	Negative	Cefotaxime	Cefotaxime	24	48	24	48
#42	Community acquired	UTI	*Klebsiella pneumoniae*	Wild type	Negative	Cefotaxime	Cefotaxime	30	54	27	51
#43	Community acquired	Pneumonia	*Escherichia coli*<br>*Citrobacter freundii*	Wild type<br>Wild type	Negative<br>Negative	Cefotaxime	Cefotaxime	24	48	24	48
#44	Healthcare associated	Pneumonia	*Klebsiella oxytoca*	Wild type	Negative	Cefotaxime	Cefotaxime	28	52	27	51
#47	Community acquired	UTI	*Escherichia coli*<br>*Proteus mirabilis*	Wild type<br>Wild type	Negative<br>Negative	Cefotaxime	Cefotaxime	30	54	26	50
#48	Community acquired	Pneumonia	*Escherichia coli*	Wild type	Negative	Amoxicilin+Clavulanate	Cefotaxime	26	49	24	47
#54	Community acquired	Peritonitis	*Escherichia coli*	Wild type	Negative	Cefotaxime	Cefotaxime	37	61	26	50
#58	Community acquired	Peritonitis	*Escherichia coli*	Wild type	Negative	Cefotaxime	Cefotaxime	48	60	44	56
Inappropriate empirical beta-lactam confirmation											
#56	Community acquired	UTI	*Escherichia coli*	Acquired cephalosporinase overproduction	***Negative***	Cefotaxime	Cefotaxime	37	60	31	54
