# Supplementary material for: Evaluation of early antimicrobial therapy adaptation guided by the BetaLACTA® test: a case-control study
Source: Crit Care. 2017 Jun 28;21:161. doi: 10.1186/s13054-017-1746-6 (PMC5488410; doi:10.1186/s13054-017-1746-6)
Supplement: Supplementary file 4 — (Predictors of an appropriate antimicrobial therapy) and Table S3. (Predictors of an optimal antimicrobial therapy). (DOCX 21 kb) [file 13054_2017_1746_MOESM4_ESM.docx]

**Table S2. Predictors of an appropriate antimicrobial therapy**

|  | **Bivariate Analysis** | |  | **Multivariate Analysis** | |
| --- | --- | --- | --- | --- | --- |
| **Variables** | OR (95% CI) | p |  | OR (95% CI) | p |
|  |  |  |  |  |  |
| Age *(per year)* | 1.00 (0.95-1.04) | 0.86 |  | - |  |
| SAPS2 *(per point)* | 1.01 (0.98-1.04) | 0.75 |  | - |  |
| Immunosuppression | 0.83 (0.28-2.63) | 0.74 |  | - |  |
| Recent travel in ESBL-endemic region | 0.32 (0.06-2.38) | 0.21 |  | - |  |
| ICU length of stay before inclusion | 0.99 (0.94-1.07) | 0.70 |  | - |  |
| Associated bacteraemia | 0.28 (0.09-0.94) | 0.03 |  | 0.28 (0.08-1.01) | 0.048 |
| Septic shock *(vs. sepsis or severe sepsis)* | 0.76 (0.25-2.42) | 0.63 |  | - |  |
| Healthcare-associated infection *(vs. community acquired)* | 0.55 (0.16-1.65) | 0.30 |  | - |  |
| Pneumonia *(vs. other sites)* | 1.23 (0.36-3.75) | 0.73 |  | - |  |
| GNB on microscopic Gram-staining examination | 0.67 (0.15-2.30) | 0.56 |  | - |  |
| Use of the betaLACTA^®^ test | 17.9 (3.4-329.7) | 0.006 |  | 18.0 (3.36-333.8) | 0.006 |

**Table S3. Predictors of an optimal antimicrobial therapy**

|  | **Bivariate Analysis** | |  | **Multivariate Analysis** | |
| --- | --- | --- | --- | --- | --- |
| **Variables** | OR (95% CI) | p |  | OR (95% CI) | p |
|  |  |  |  |  |  |
| Age *(per year)* | 1.00 (0.96-1.03) | 0.78 |  | - |  |
| SAPS2 *(per point)* | 0.99 (0.97-1.01) | 0.33 |  | - |  |
| Immunosuppression | 1.05 (0.46-2.45) | 0.91 |  | - |  |
| Recent travel in ESBL-endemic region | 0.49 (0.10-2.61) | 0.37 |  | - |  |
| ICU length of stay before inclusion | 0.99 (0.95-1.05) | 0.79 |  | - |  |
| Associated bacteraemia | 0.53 (0.20-1.40) | 0.19 |  | 0.41 (0.10-1.60) | 0.21 |
| Septic shock *(vs. sepsis or severe sepsis)* | 0.79 (0.35-1.84) | 0.58 |  | - |  |
| Healthcare-associated infection *(vs. community acquired)* | 0.55 (0.24-1.22) | 0.15 |  | 0.42 (0.15-1.10) | 0.08 |
| Pneumonia *(vs. other sites)* | 0.99 (0.40-2.33) | 0.99 |  | - |  |
| GNB on microscopic Gram-staining examination | 0.65 (0.24-1.63) | 0.38 |  | - |  |
| Use of the betaLACTA^®^ test | 32.6 (9.0-209.7) | <0.001 |  | 35.5 (9.6-231.9) | <0.001 |
