# Supplementary figures and images for: Evaluation of early antimicrobial therapy adaptation guided by the BetaLACTA® test: a case-control study
Source: Crit Care. 2017 Jun 28;21:161. doi: 10.1186/s13054-017-1746-6 (PMC5488410; doi:10.1186/s13054-017-1746-6)

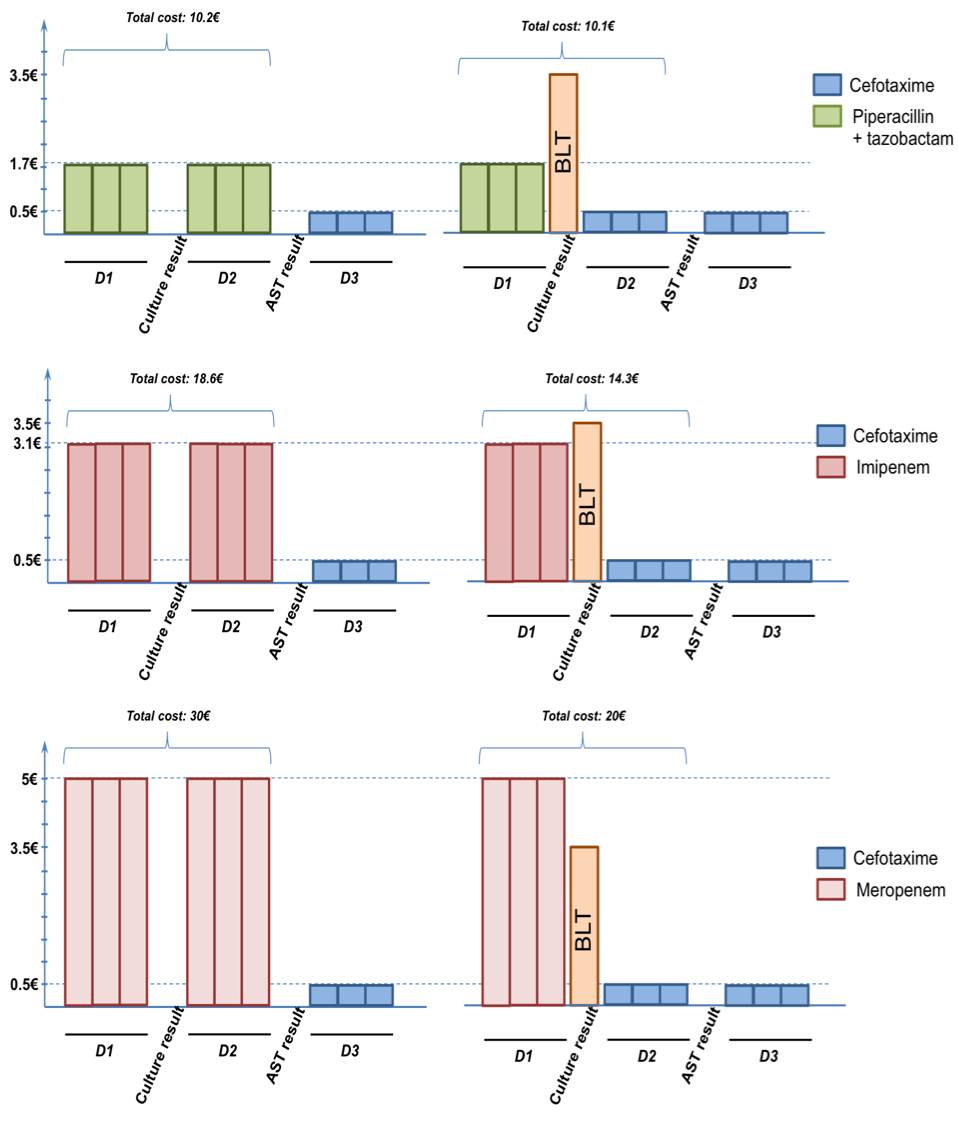

Supplement: Supplementary file 5 — Comparison of the antimicrobial therapy-related costs between the conventional strategy (left) and the BLT-guided strategy (right) for de-escalation from piperacillin-tazobactam (first line), imipenem (middle line) and meropenem (last line) to cefotaxime. Given antimicrobial prices in our structure and considering antimicrobial administration three times a day, BLT-guided de-escalation from piperacillin-tazobactam to cefotaxime costs the same as a 48-hour empirical administration of piperacillin-tazobactam, while BLT-guided de-escalation from carbapenem to cefotaxime is cheaper than a 48-hour empirical administration of imipenem (-4€) or meropenem (-10€). (PNG 160 kb) [file 13054_2017_1746_MOESM5_ESM.png]
